# Predictive validation of the repeated low-dose reserpine rodent model of parkinsonism

**DOI:** 10.1007/s00221-026-07316-8

**Published:** 2026-05-11

**Authors:** Vinicius Bioni, Alvaro C. Lima, Debora M. G. Cunha, Narriman Gonçalves, Leonardo B. Lopes-Silva, João P. Kurita, Manuela B. L. Soares, Marcela Becegato, Aline C. Ramos, Anderson H. F. F. Leão, Regina H. Silva

**Affiliations:** 1 Department of Pharmacology, Universidade Federal de São Paulo, São Paulo, Brazil; 2 Department of Psychiatry, Universidade Federal de São Paulo, São Paulo, Brazil

**Keywords:** L-DOPA, Parkinson’s disease, Animal model, Progressive neurodegeneration, Dopamine depletion

## Abstract

L-DOPA (LD) is the gold-standard treatment of motor symptoms in Parkinson’s disease (PD), conventionally used to prove the predictive value of PD animal models. This study aimed to investigate the predictive validity of an adaptation of the conventional reserpine model of PD: the repeated low-dose reserpine administration in rodents, which promotes progressive motor impairment in catalepsy, vacuous chewing behavior tests, and neurochemical deficits. Specifically, we investigated the effects of acute and chronic treatment with LD on motor alterations induced in mice by this modified protocol. Swiss mice were treated with 20 reserpine injections (0.1 mg/kg, s.c., every other day). Acute LD (25, 50, 100, and 200 mg/kg) administration was performed at the end of reserpine protocol to assess immediate motor recovery. Additionally, concomitant chronic LD (50 and 100 mg/kg) treatment was conducted to investigate potential attenuation of motor impairment and dopamine depletion quantified by HPLC. Repeated reserpine protocol induced motor deficits in catalepsy and vacuous chewing evaluations and striatal dopamine depletion. Acute LD administration significantly improved catalepsy in higher doses, while chronic LD treatment attenuated motor impairments and reduced dopamine depletion in the striatum. These findings support the predictive validity of the reserpine rodent model for studying parkinsonism. Together with the ability to promote chronic and progressive parkinsonian alterations, the reversion of these deficits by LD reinforces the potential of the repeated low-dose reserpine protocol for studies of potential therapies and the pathophysiology underlying neurodegenerative processes in PD.

## Introduction

Parkinson’s disease (PD) is a neurodegenerative disorder characterized by motor impairments, with increased incidence in aging populations (Lau and Breteler 2006; Silva et al. 2024). The clinical diagnosis is based on the observation of cardinal motor signs, including akinesia, bradykinesia, rigidity, and resting tremor (Agnello et al. 2024).

The onset and severity of motor signs are associated with the progressive loss of the substantia nigra (SN) dopaminergic neurons, leading to dopamine (DA) depletion in the dorsal striatum (STR) (Wirdefeldt et al. 2011). Additional alterations include increased levels of oxidative stress, defects in cell respiratory chain, neuroinflammation, and the accumulation of protein aggregates known as Lewy bodies, mainly constituted by alpha-synuclein and ubiquitin proteins (Subramaniam and Chesselet 2013; Kalia and Lang 2015; Cunha et al. 2022; Cohen et al. 2024; Macedo et al. 2025).

Dopamine replacement with L-DOPA (LD) has been the most effective treatment for motor deficits in PD for a long time (Hauser 2009; Antonini et al. 2025; Mittal et al. 2025). However, long-term treatment with LD can result in disabling side effects, such as LD-induced dyskinesia (LID) (Gottwald and Aminoff 2011; Lane 2019), psychosis(Jankovic and Aguilar 2008), and anxiety (Prediger et al. 2012). Moreover, its efficacy diminishes over time, probably due to the progressive loss of the dopaminergic neurons (Lane 2019; Berg et al. 2012; Barriere et al. 2025). Consequently, LD therapy usually starts under a low dose and/or in combination with other agents such as dopamine agonists or monoamine oxidase inhibitors (Grandas et al. 1999).

Reserpine is an irreversible vesicular monoamine transporter (VMAT) inhibitor, and the administration of this drug was the first pharmacological animal model of PD in rodents (Silva et al. 2024; Leao et al. 2015; Carlsson et al. 1957). Early studies administered acute high doses (1 mg/kg or over) of reserpine to induce severe motor deficits and predict the efficacy of pharmacological treatments in alleviating the motor disturbances (Carlsson et al. 1957; Goldstein et al. 1975), including LD (Carlsson et al. 1957). Besides the motor deficit related to monoamine vesicle depletion, posterior studies also showed that reserpine was potentially toxic to the neurons by inducing oxidative stress (Abilio et al. 2003, 2004, 2002; Faria et al. 2005).

Over time, acute reserpine administration was replaced by neurotoxic models employing neurotoxins such as 6-hydroxydopamine (6-OHDA), 1-methyl-4-phenyl-1,2,3,6-tetrahydropyridine (MPTP), and rotenone. While these models also have limitations (Jagmag et al. 2015; Leal et al. 2016), their ability to induce dopaminergic neuronal death is considered an important feature. Nevertheless, both acute high-dose reserpine and classic neurotoxin (6-OHDA and MPTP) treatments induce short-term severe motor impairment, which precludes the study of progressive behavioral (motor and non-motor) and neuronal alterations across PD development (Jenner 1989; Jeon et al. 1995; Duty and Jenner 2011; Kozina et al. 2014; Ma et al. 2014).

In the early 2010s, our research group proposed a repeated low-dose reserpine treatment protocol (0.1 mg/kg, every other day) in rats (Fernandes et al. 2012), which was subsequently replicated in mice (Brandao et al. 2017; Campelo et al. 2017; Lopes-Silva et al. 2024; Soares et al. 2021). The protocol allows for the assessment of motor, non-motor, and neurochemical impairments across repeated treatment simulating the progression of these changes in PD (Silva et al. 2024; Lopes-Silva et al. 2024; Santos et al. 2013).

As originally proposed by Willner (Willner 1991), animal models in neuropsychiatric diseases should meet criteria for face, construct, and predictive validity. The low-dose repeated reserpine rodent model of parkinsonism has demonstrated solid evidence of face and construct validity. Face validity was shown by progressive (1) difficulty to start movement evaluated by the catalepsy test; (2) reduced spontaneous activity in the open field; (3) increased oral dyskinesia; (4) balance impairment and (5) non-motor signs that emerge before motor alterations, such as short-term recognition deficits, anxiety and nociceptive changes (Fernandes et al. 2012; Santos et al. 2013). Construct validity includes mainly neurochemical alterations in the nigrostriatal pathway across repeated low-dose reserpine treatment: (1) decrement of tyrosine hydroxylase (TH) (Santos et al. 2013; Leao et al. 2017; Lima et al. 2021); (2) decreased immunostaining of brain derived neurotrophic factor (BDNF) (Campelo et al. 2017); (3) reduced dopamine levels (Leao et al. 2017); (4) increased lipid peroxidation (Fernandes et al. 2012; Leao et al. 2017; Olivatto et al. 2024), (5) increased alpha-synuclein expression (Leao et al. 2017; Beserra-Filho et al. 2022) and (6) presence of neuroinflammation markers (Cunha et al. 2022). In addition, both behavioral and neuronal deficits were aggravated by age (Melo et al. 2022) or in the male sex (Lima et al. 2021), which matches the higher prevalence of the disease in aged individuals and men, respectively.

In contrast, predictive (or pharmacological) validity of the repeated reserpine protocol has only been demonstrated for non-standard interventions, focusing on prevention or delay of neurodegeneration, such as environmental enrichment (Campelo et al. 2017) and treatment with classical anti-oxidant agents (Sarmento-Silva et al. 2014) or natural extracts with neuroprotection potential (Brandao et al. 2017; Beserra-Filho et al. 2022, 2019; Lins et al. 2018; Silva-Martins et al. 2021). The repeated low-dose reserpine protocol has been particularly useful for assessing interventions that delay disease progression, an outcome that cannot be observed in acute models (Silva et al. 2024). However, the efficacy of standard symptomatic LD therapy in reducing motor deficits and dopamine depletion within this model remains to be investigated.

Thus, we aimed to improve the validation of this protocol as a model to study PD by investigating the effects of acute and chronic LD in mice submitted to the low-dose repeated reserpine treatment.

## Material and methods

### Animals

Seven-month-old male Swiss mice were used in this study. All animals were randomly housed in groups of 4–5 per cage (30 × 20 × 12 cm) under controlled conditions of airflow and temperature (23 ± 1 °C), with a 12 h light/12 h dark cycle (lights on 6:30 a.m.). Food and water were available ad libitum. Animals used in this study were handled following the guidelines of the Brazilian law for the use of animals in research. All procedures were approved by the local ethics committee (CEUA-UNIFESP-6415270417/2017) and aimed to minimize animal pain, suffering, or discomfort.

### Drugs

Reserpine (RES, Sigma Chemical Co. St. Louis, MO) was dissolved in 1% glacial acetic acid, diluted to the correct concentration in distilled water and given at a dose of 0.1 mg/kg. Vehicle (Veh) consisted of the same amount of acetic acid and distilled water as in the reserpine solution. These solutions were injected subcutaneously (s.c.).

L-DOPA/Carbidopa (LD; proportion 10:1, doses are described below; Sigma Chemical Co., St. Louis, MO) combination was diluted to the correct concentration in saline solution. Saline was used as a control solution. These drugs were injected intraperitoneally (i.p.). Doses are specified below, according to the experimental design.

All solutions were administered at a volume of 10 ml/kg of body weight.

### Experimental design

#### Experiment I—effects of acute L-DOPA on motor deficits induced by repeated low-dose reserpine.

**Fig. 1:**
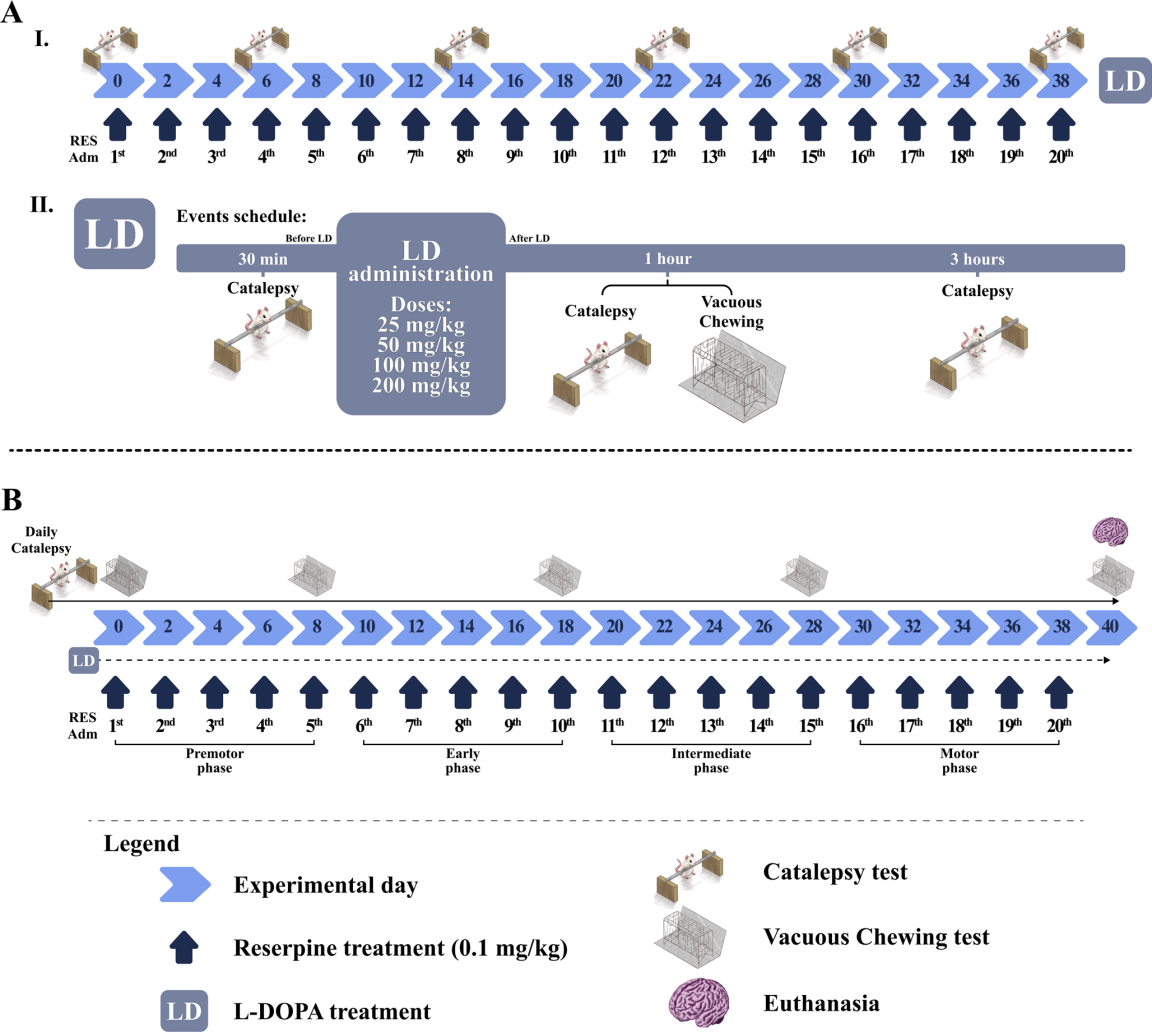
Experimental designs of experiments I (**A**) and II (**B**). Symbols: Light blue blocks represent experimental day, dark blue arrows represent reserpine injections on alternate days, continuous arrows are the sequence of behavioral tests, long dash diamond point represents daily LP administration, crescent moon represents catalepsy test, pointed arrow represents the daily catalepsy test, four-point star represents vacuous chewing test

Forty-nine animals were randomly distributed into 6 groups (*n* = 8–9 per group): (1) Vehicle-Saline (VS); (2) Reserpine-Saline (RS); (3) Reserpine-L-DOPA 25 mg/kg (RL25); (4) Reserpine-L-DOPA 50 mg/kg (RL50); (5) Reserpine-L-DOPA 100 mg/kg (RL100); and (6) Reserpine-L-DOPA 200 mg/kg (RL200). Animals received 20 injections of vehicle or reserpine (0.1 mg/kg) on alternate days, according to the treatment group. Catalepsy behavior was evaluated at baseline and every 4 injections of reserpine (1st, 2nd, 3rd, 4th, and 5th measurements), as illustrated in Fig. 1A (I).

Forty-eight hours after the last reserpine injection, another catalepsy (6th measurement) was performed before the acute LD or saline administration. One hour after acute treatment with LD, animals were submitted to the following motor evaluations: (1) catalepsy test (7th measurement); (2) vacuous chewing assessment, and a final (3) catalepsy test (3 h after the LD administration, 8th measurement), as illustrated in Fig. 1A (II).

#### Experiment II—effects of chronic L-DOPA on motor deficits induced by repeated low-dose reserpine

Another set of fifty-four animals were randomly distributed into 6 groups (*n* = 8 per group): (1) Vehicle-Saline (VS); (2) Vehicle-L-DOPA 50 mg/kg (VL50); (3) Vehicle-L-DOPA 100 mg/kg (VL100); (4) Reserpine-Saline (RS); (5) Reserpine-L-DOPA 50 mg/kg (RL50); and (6) Reserpine-L-DOPA 100 mg/kg (RL100). Animals received 20 injections of vehicle or reserpine (0.1 mg/kg, every other day) concomitant to 40 daily injections of LD (50 or 100 mg/kg) or saline, according to the treatment group. The LD doses used in this experiment were chosen based on the results from experiment I.

Throughout the experiment, animals were submitted to the following tests: (1) daily catalepsy tests; and (2) vacuous chewing assessment 48 h after the 5ᵗʰ, 10ᵗʰ, 15ᵗʰ, and 20ᵗʰ reserpine injections. The last assessments were performed 48 h after the last reserpine injection. The experimental design is illustrated in Fig. 1B.

### Motor evaluation

#### Catalepsy test

**Fig. 2:**
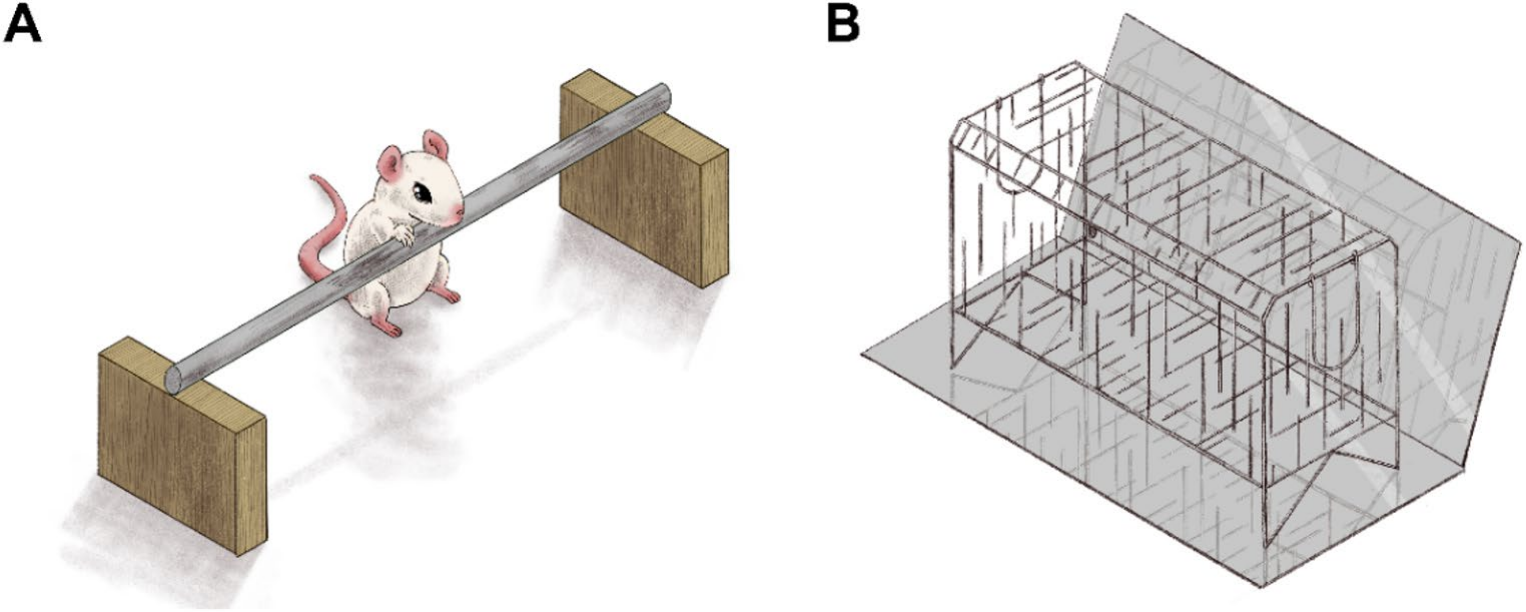
Illustration of the catalepsy test (**A**) and vacuous chewing assessment (**B**) experimental sets

The catalepsy test was conducted by placing the animal’s forepaws on a horizontal bar positioned 5 cm above the bench surface. Catalepsy was defined as an immobile posture, keeping both forepaws on the bar (illustrated in Fig. 2A). Latency to remove at least one paw from the bar was measured up to a maximum of 180 s. Three trials were carried out for each animal on each observation day, and the results were analyzed considering the mean value of the three trials (Lopes-Silva et al. 2024; Beserra-Filho et al. 2019; Custodio-Silva et al. 2024).

The catalepsy analysis for acute LD treatment (experiment I) was conducted in two steps: (1) comparing the reserpine and vehicle throughout the protocol, with each measure corresponding to 5 reserpine injections, and (2) comparing catalepsy across different time points and LD treatments.

For chronic LD treatment (experiment II), catalepsy analysis was performed in sections, with each section consisting of 10 observation days (equivalent to five reserpine injections). The sections were defined as follows: basal (no substance administration), premotor (1^st^–5th injection), early motor (6^th^–10th injection), intermediate motor (11th–15th injection), and motor (16^th^–20th injection) phases.

#### Vacuous chewing movements

The mice were individually placed in a wired cage (30 cm × 15 cm × 19 cm) with mirrors positioned under the floor and behind the back wall of the cage to allow behavioral quantification when the animal faced away from the observer (as illustrated in Fig. 2B). The frequency of vacuous chewing movements (mouth openings in the vertical plane not directed toward physical material) was measured continuously for 10 min.

### HPLC (high performance liquid chromatography)

For the HPLC essay, only animals from concomitant LD administration were used. At the end of the chronic protocol (48 h after the last reserpine administration), animals were euthanized by decapitation, brains were rapidly removed, immediately frozen in isopentane, and stored at − 80 °C until dissection. Striatal tissue samples were collected using a cryostat (Leica CM1890) maintained at − 25 °C, guided by a mouse brain atlas (Franklin and Paxinos, 2007). Bilateral striatal punches were obtained with a stainless-steel cannula of 500 μm inner diameter. Two or three punches per animal were sampled from 50-μm-thick coronal sections spanning the entire rostrocaudal extent of the striatum. The punches were transferred into pre-weighed microtubes, which were then re-weighed on a precision balance to record tissue mass and immediately stored at − 80 °C. The striatal region was identified by anatomical landmarks, beginning at the level immediately caudal to the opening of the corpus callosum and extending to the section where the hippocampal formation first becomes apparent, corresponding to the bregma coordinates between + 1.34 mm and − 0.10 mm.

On the day of the biochemical analysis, tissues were homogenized in perchloric acid with isoproterenol, as control molecules, proportional to tissue weight. Samples were homogenized and put to rest for 30 min on ice and centrifuged at 14,000 rpm for 15 min at 4 °C. Supernatants were filtered and aliquoted, pH adjusted to 3 and then injected into the auto-sampler plaque to EiCOM-HPLC analysis system.

The tissue levels of DA were measured by an HPLC-ECD system. HPLC column (Octadecyl silyl SC-3ODS column (3 μm, 100 mm × 3 mm, EiCOM®), preceded by an AC-ODS precolumn (ID 3.0 × 4.0 mm), was used. The mobile phase consisted of 80% 0.1 M citrate-acetate buffer (pH 3.5), 20% methanol, 220 mg/l sodium octane sulfonate (SOS), and 5 mg/l EDTA-2Na. The potential of the electrode was set at + 750 mV. The electrochemical signals were recorded via an interface by a computer equipped with the EiCOM Envision EPC-700 version 1.0b.

### Statistical analysis

Data normality and homogeneity of variances were tested by Shapiro–Wilk and Levene’s tests, respectively.

For experiment 1, the catalepsy test was performed by one-way repeated measures analysis of variance (ANOVA). In the last day (acute L-DOPA injection), the catalepsy and vacuous chewing were analyzed by one-way ANOVA, with treatment (vehicle/reserpine and saline/L-DOPA) as a between-subject factor, followed by Sidak’s or Dunnett’s post hoc test.

For experiment 2, catalepsy and vacuous chewing were analyzed by two-way repeated measures ANOVA (considering time and both treatments as factors), followed by Sidak’s post hoc test. HPLC analysis was performed by Mann–Whitney non-parametric analysis. A non-parametric correlation between DA levels and the last catalepsy measure was performed by Spearman’s rho test.

Results were expressed as mean + SEM (parametric analyses) or median with max and minimum values (non-parametric analyses), *p* ≤ 0.05 was considered to reflect significant differences. All statistical analyses were conducted using SPSS (IBM, USA) and Prism (GraphPad, USA).

## Results

### Acute L-DOPA treatment (experiment I)

#### Catalepsy tests

In experiment 1, one-way repeated measures ANOVA was conducted in two steps: (1) comparing the vehicle and reserpine protocols across time, and (2) addressing the effect of acute LD treatment.

**Fig. 3:**
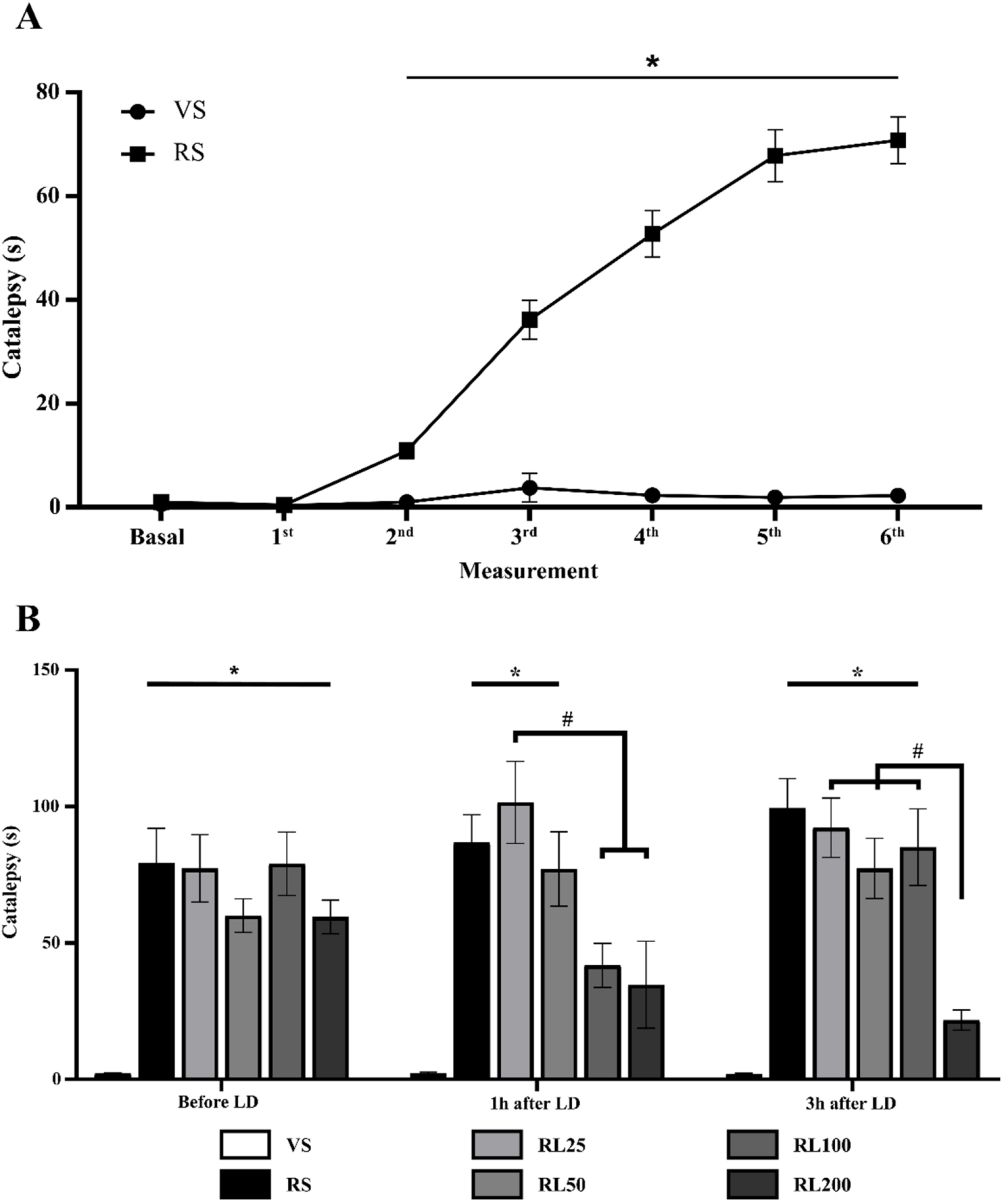
Catalepsy duration across reserpine treatment (each measurement corresponds to 4 reserpine injections) (**A**); and effect of L-DOPA (L) acute administration in different moments (before, after 1 h and 3 h of L-DOPA administration), after the last reserpine injection (**B**). *p ≤ 0.05 compared to Vehicle (VS-Dunnett’s post hoc test); ^#^*p* ≤ 0.05 compared to L-DOPA groups (Sidak’s post hoc test). Data are expressed as means ± SEM (*n* = 8)

The comparison of animals in vehicle/reserpine group was conducted across time (from basal until the 6th measure) by repeated measures ANOVA with treatment as a between-subject factor. The analysis demonstrated an effect of time [F (6, 282) = 25.951; *p* < 0.001], reserpine treatment [F (1, 47) = 36.715; *p* < 0.001], and the interaction between time and reserpine treatment F (6, 282) = 23.507; *p* < 0.001]. Sidak’s post hoc revealed that the reserpine group had a motor impairment, i.e., increased catalepsy duration after the 2nd measurement (after 3 reserpine administrations; *p* < 0.007), until the 6th measurement (after 19 reserpine administrations; *p* < 0.001), as demonstrated in Fig. 3A.

For the evaluation of the effect of acute LD, only the reserpine groups were compared. A one-way repeated measures ANOVA was applied comparing the LD treatment (saline, 25, 50, 100 and 200 mg/kg) across time (before, after 1 and 3 h L-DOPA injection), as shown in Fig. 3B. These analyses demonstrate the effect of LD treatment [F (4,36) = 5.839; *p* < 0.001] and the interaction between LD treatment and time (before and 1 and 3 h after injection) [F (8, 72) = 4.178; *p* < 0.001]. Dunnet’s post hoc compared all groups with VS (vehicle saline), demonstrating a decrease on the catalepsy time after 1 h (RL100 and RL200; *p* = 0.101 and 0.206, respectively) and 3 h of LD administration at the dose of 200 mg/kg, (*p* = 0.439).

Sidak’s post hoc comparison among reserpine groups (RS, RL25, RL50, RL100, and RL200) revealed that there was a decrease in the catalepsy time shown by the RL25 group compared to RL100 and RL200 (*p* = 0.030 and 0.008, respectively) after 1 h of LD administration. In addition, RL200 showed a tendency to decrease the catalepsy time in comparison to the Saline group (*p* = 0.069). After 3 h of LD administration, Sidak’s post hoc showed a decrease in time on the catalepsy test in RL200 compared with RS (*p* < 0.001), RL25 (*p* < 0.001), RL50 (*p* = 0.005), and RL100 (*p* = 0.001) groups.

#### Vacuous chewing movements

**Fig. 4:**
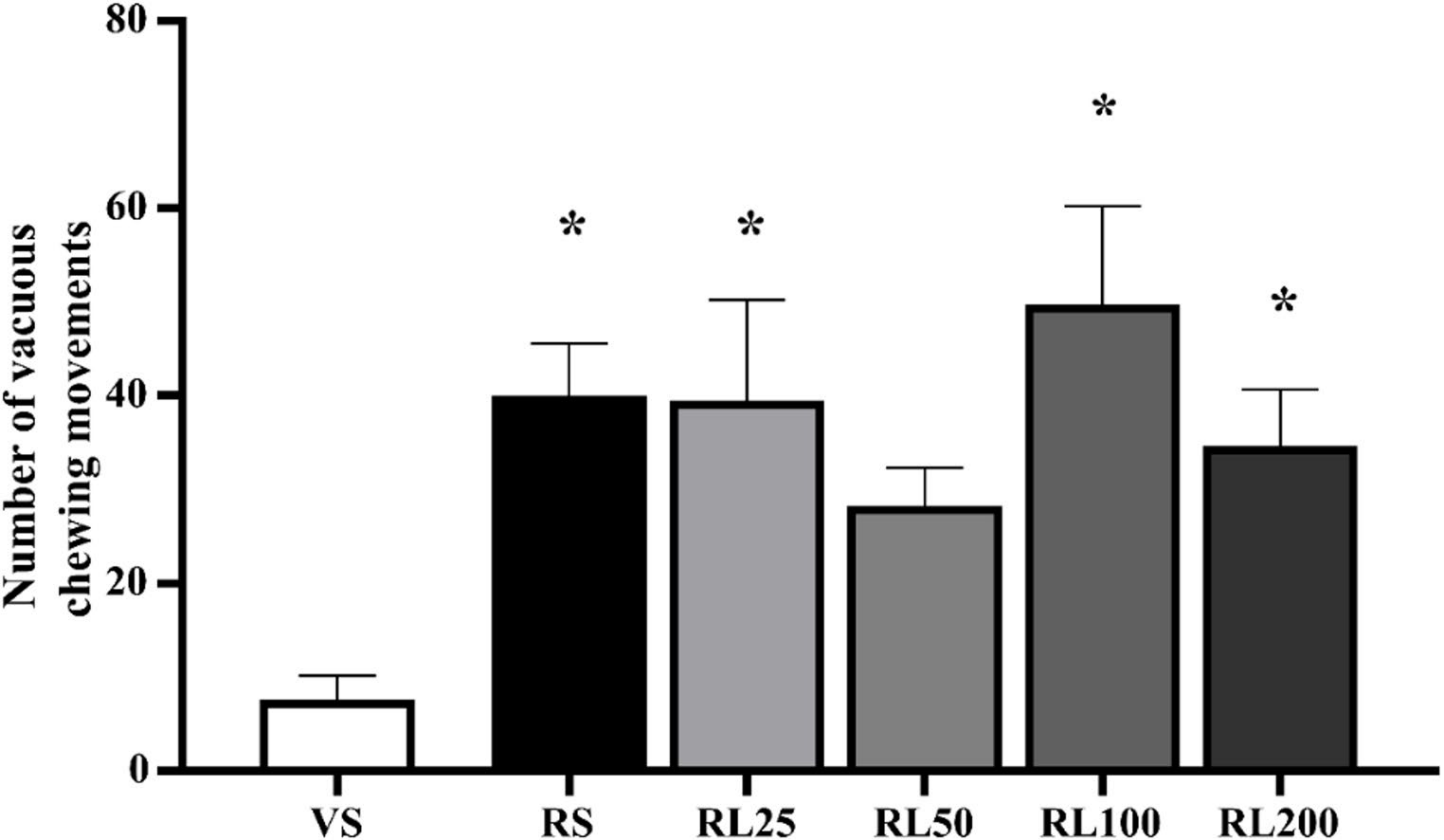
Number of vacuous chewing movements after 1 h of L-DOPA (L) injections (Sal, 25, 50, 100 and 200 mg/kg) in the reserpine (R) protocol. *p ≤ 0.05 compared to Vehicle (VS—one-way ANOVA followed by Dunnett’s test, *p* ≤ 0.05). Data are expressed as means ± SEM (*n* = 8)

In the acute protocol, the vacuous chewing observation was performed 2 h after the LD administration. A one-way ANOVA revealed an increase in the number of vacuous chewing movements in reserpine compared to vehicle, which received saline (VS and RS) [F (1, 15) = 28.167; *p* < 0.001]. Dunnet’s post hoc indicated that all groups (except RL50, *p* = 0.183) presented more vacuous chewing in comparison with VS (RS: *p* = 0.013; RL25: *p* = 0.015; RL100: *p* = 0.001; RL200: *p* = 0.040). The results are demonstrated in Fig. 4.

### Chronic L-DOPA treatment

#### Catalepsy

**Fig. 5:**
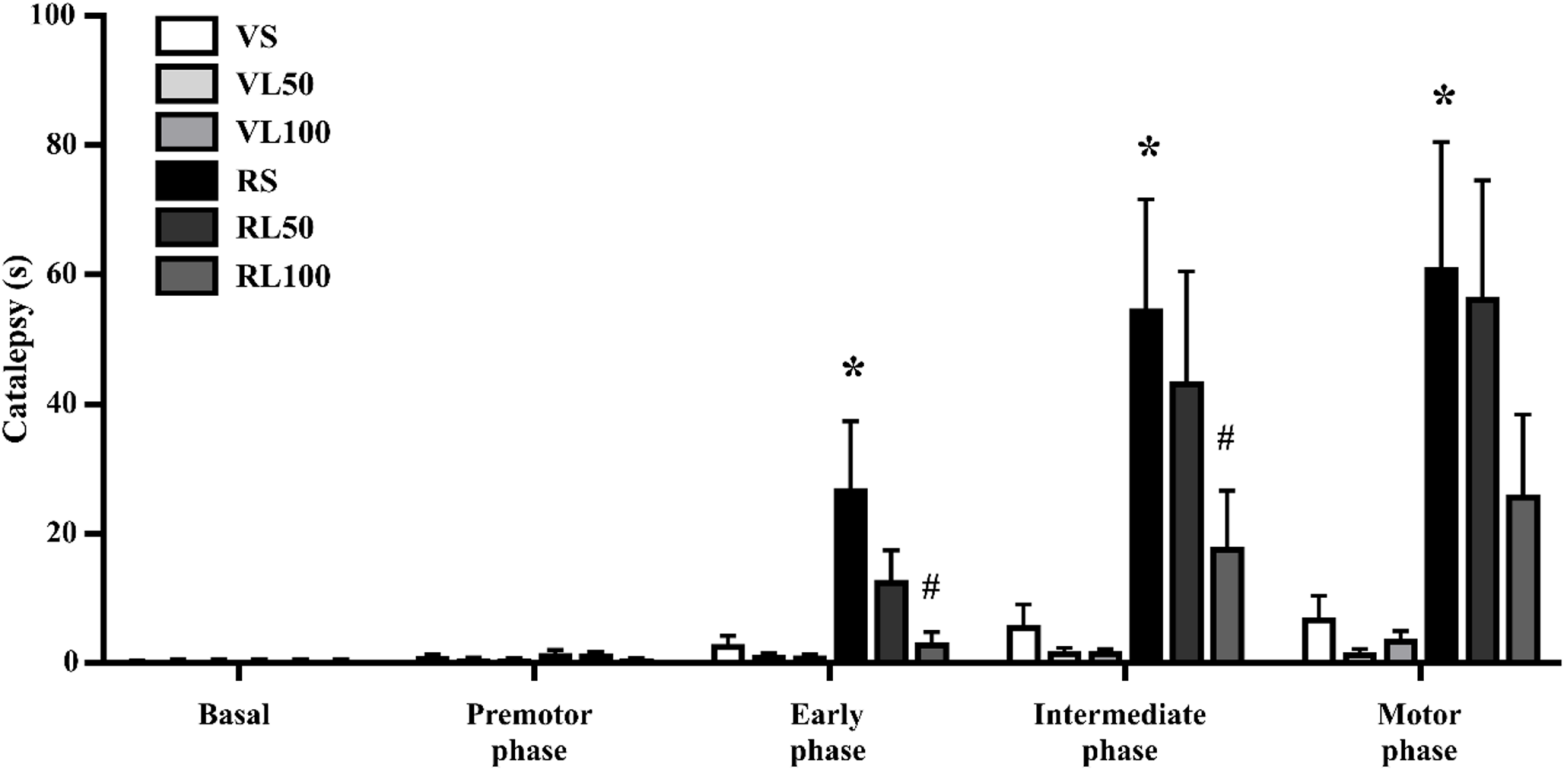
Catalepsy phases across the concomitant L-DOPA (L) and reserpine (R) protocol. Each phase represents the mean of the assessments conducted under 5 reserpine applications: basal (previously to reserpine), premotor (1st to 10th assessments), initial (11th to 20th assessments), intermediate (21st to 30th assessments), and motor (31st to 40th assessments). *p ≤ 0.05 compared to Vehicle; ^#^*p* ≤ 0.05 compared to Saline (ANOVA with repeated measures followed by Sidak’s test, *p* ≤ 0.05). Data are expressed as means ± SEM (*n* = 8–9)

The catalepsy in the LD chronic protocol was performed and analyzed in phases: Basal (without reserpine administration), premotor phase (1^st^–5th reserpine administration), initial phase (6^th^–10th reserpine administration), intermediate phase (11^th^–15th reserpine administration), and motor phase (16^th^–20th reserpine administration). A two-way repeated measures ANOVA demonstrated an effect of phases [F (4,152) = 22.302; *p* < 0.001], reserpine treatment (vehicle or reserpine) [F (1,38) = 17.076; *p* < 0.001], and the interaction between phases and reserpine treatment [F (4,152) = 16.922; *p* < 0.001], as seen in Fig. 5.

Sidak’s post hoc test revealed that the RS group spent more time on the catalepsy bar than the VS group from the initial until motor phase (*p* = 0.002, 0.003, and 0.003, respectively). The RL50 stayed longer on the catalepsy bar in intermediate (*p* = 0.009) and motor phases (*p* = 0.003). On the other hand, chronic treatment with 100 mg/kg LD prevented the increase in catalepsy at all time points. Indeed, RL100 did not differ from VL100.

Comparing the LD doses (saline, 50, and 100 mg/kg of LD), the RL100 group spent less time on the catalepsy bar than RS in the initial (*p* = 0.008) and intermediate phases (*p* = 0.050).

#### Vacuous chewing movements

**Fig. 6:**
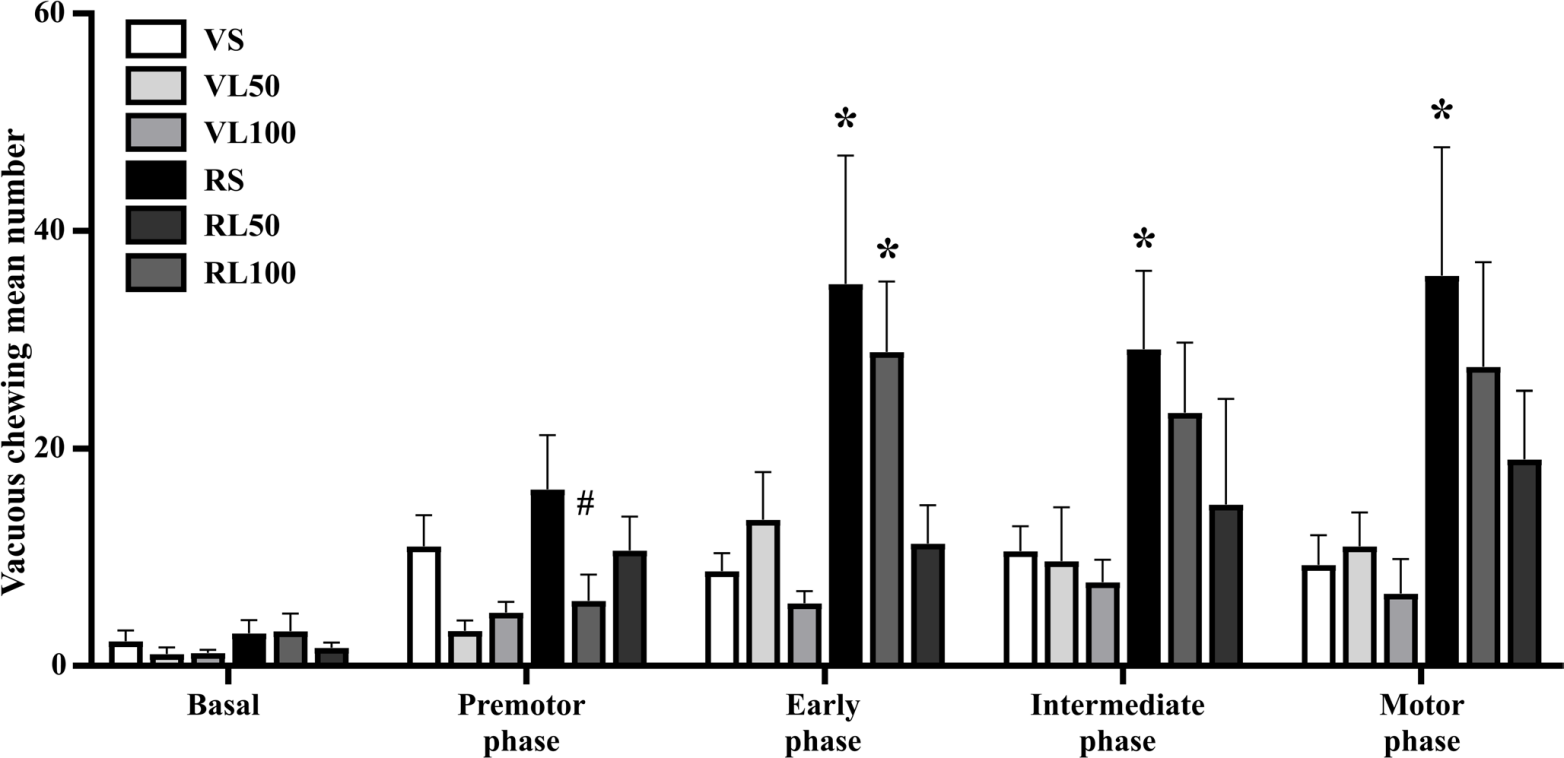
Number of vacuous chewing movements along the concomitant L-DOPA (L) and reserpine (R) protocol. *p < 0.05 compared to vehicle; ^#^*p* ≤ 0.05 compared to Saline (ANOVA with repeated measures followed by Sidak’s test, *p* ≤ 0.05). Data are expressed as means ± SEM (*n* = 8–9)

The evaluation of vacuous chewing in the chronic protocol was conducted before any drug administration (Basal), after the 5th (premotor phase), 10th (early phase), 15th (intermediate phase), and 20th reserpine injection (motor phase). Two-way repeated measures ANOVA revealed an effect across time [F (4,140) = 15.874; *p* < 0.001], effect of reserpine treatment [F (1,35) = 10.436; *p* = 0.003], and the effect of reserpine across time [F (4,140) = 5.368; *p* = 0.003], demonstrated in Fig. 6.

Sidak’s post hoc test revealed that the reserpine/saline (RS) group increased the vacuous chewing number over time, from the early phase (*p* = 0.006; 0.040; 0.011, respectively). The reserpine/L-DOPA 50 mg/kg (RL50) group performed less vacuous chewing at the premotor phase in comparison to the RS group (*p* = 0.049).

#### HPLC analyses

**Fig. 7:**
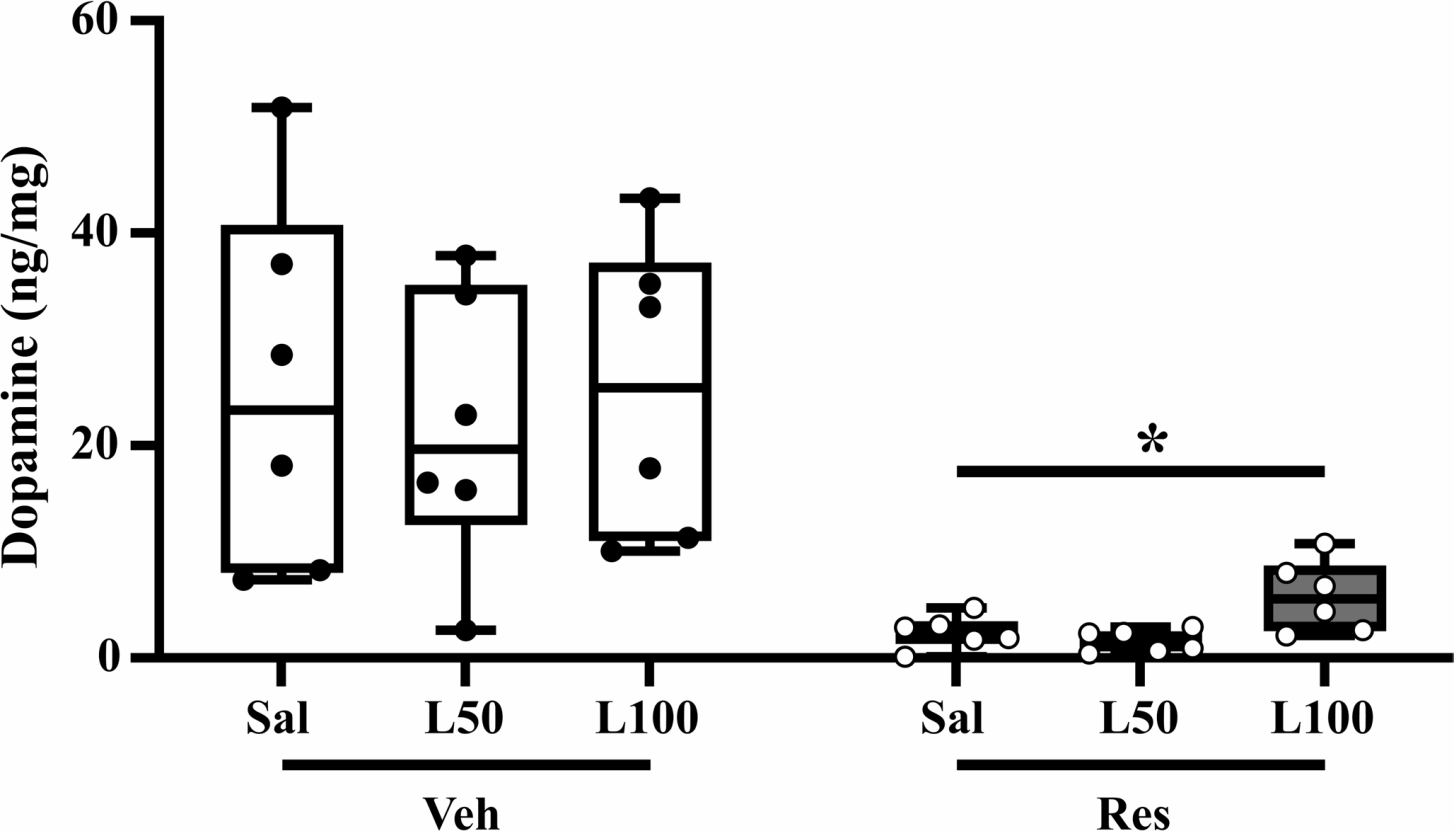
Dopamine quantification by HPLC, in fresh frozen brain obtained forty-eight hours after the last reserpine injection in experiment II. Circles indicate individual data in each group. *p ≤ 0.05 compared to Vehicle (Mann–Whitney test). Data are expressed as median with max and minimum values (*n* = 6)

For dopamine quantification in the striatum, the Mann–Whitney test compared vehicle and reserpine for each LD treatment, showing a decrease in dopamine quantification in all L-DOPA doses, saline (U = 0.000; *p* = 0.04), L50 (U = 0.000; *p* = 0.06), and L100 (U = 0.000; *p* = 0.06), as demonstrated in Fig. 7.

**Fig. 8:**
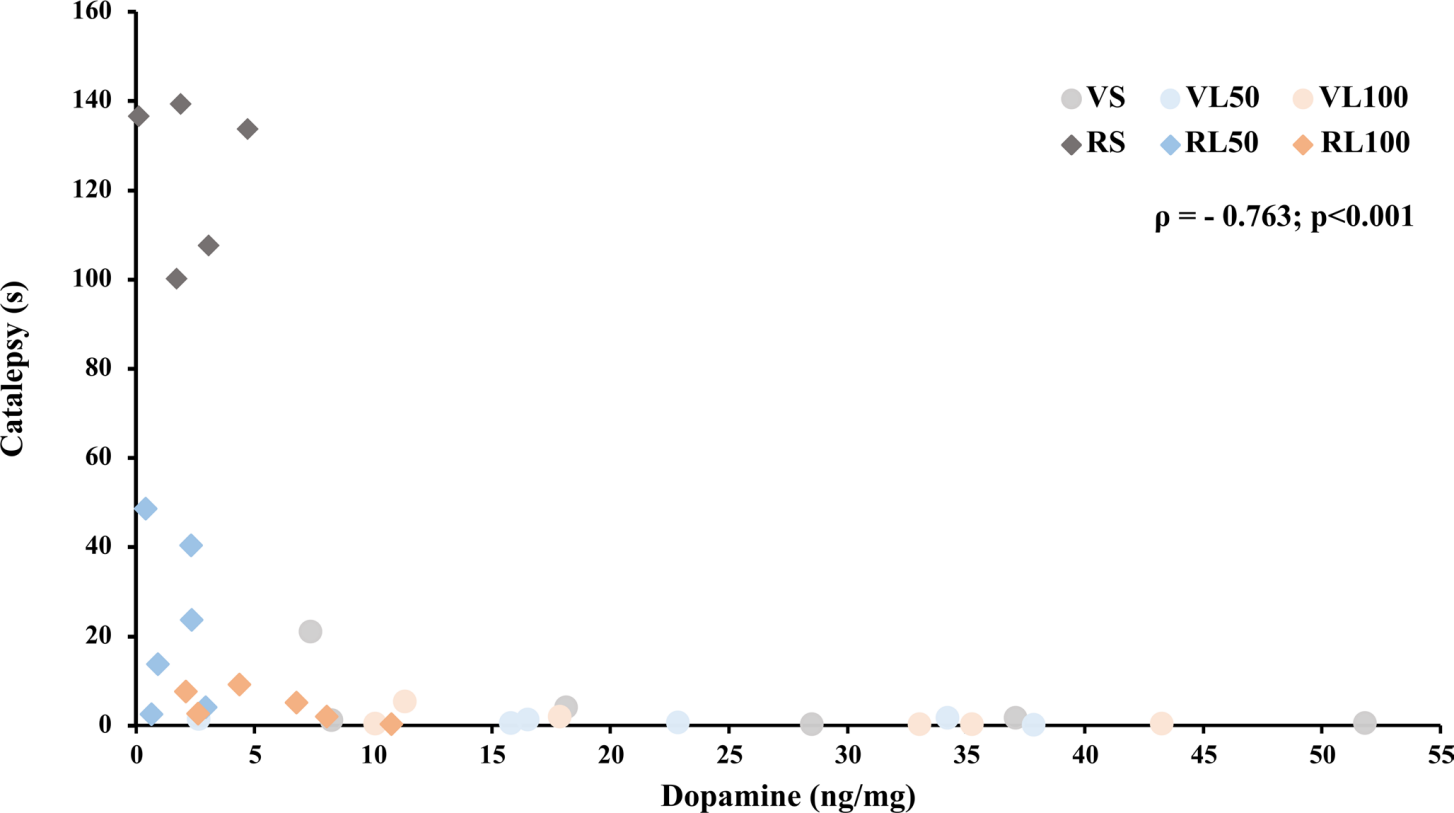
Catalepsy and Dopamine quantification correlation (Spearman’s rho). Circles indicate vehicle groups and diamonds represent reserpine groups (*n* = 5–6)

Spearman’s test showed a negative correlation between DA quantification and the duration of catalepsy in the last test (*ρ *= − 0.763**; p < 0.001), which is demonstrated in Fig. 8.

## Discussion

In this study, we investigated the predictive validity of a progressive animal model for PD induced by repeated low doses of reserpine, assessing the effects of acute and chronic LD treatment. Repeated low doses of RES resulted in increased cataleptic behavior and increased vacuous chewing movements, resembling alterations seen in patients, such as akinesia, hypokinesia, resting tremor, and dyskinesias. In addition, this protocol resulted in striatal dopamine depletion, which was either reversed or attenuated by acute and chronic LD treatment.

Previous studies have shown that catalepsy and dyskinesia are related to monoamine depletion in the striatum and substantia nigra, regions critically involved in the motor symptoms of PD (Colpaert 1987; Neisewander et al. 1994; Heeringa and Abercrombie 1995; Lima et al. 2025). Consistent with these findings, we observed that chronic treatment with a low dose of reserpine resulted in progressive behavioral impairment associated with striatal dopamine depletion, supporting earlier reports (Fernandes et al. 2012; Leao et al. 2017; Shen et al. 2025; Santos et al. 2015; Salamone and Baskin 1996). Acute LD treatment significantly reduced reserpine-induced catalepsy, while chronic administration prevented or delayed behavioral deterioration in a dose-dependent manner. Notably, the highest LD dose was also able to partially restore the striatal dopamine levels.

Both acute and chronic protocols of reserpine administration have been shown to increase oral movements in rodents (Abilio et al. 2004; Fernandes et al. 2012; Leao et al. 2017; Lima et al. 2021, 2025; Beserra-Filho et al. 2022; Silva et al. 2002). Although the mechanisms underlying this behavior remain unclear, both post-synaptic DA receptors sensitization (LaHoste and Marshall 1994) and striatal oxidative stress (Abilio et al. 2004) have been proposed as neuronal alterations related to reserpine-induced oral dyskinesia. Nevertheless, increases in oral movements in rodents are considered analogous to parkinsonian tremors and have been used to study the involvement of neurotransmitters in the pathophysiology of PD (Salamone and Baskin 1996; Carlson et al. 2003; Salamone et al. 1998; Wang et al. 2025). However, in our study, the acute LD did not modify reserpine-induced oral movements. On the other hand, higher dopamine availability in chronically treated animals prevented the increase in vacuous chewing movements. This effect may be attributed to sustained dopamine levels, which likely prevented compensatory plastic changes in dopamine receptors triggered by repeated reserpine-induced dopamine depletion. Indeed, our reserpine protocol has been shown to induce dopaminergic sensitization, as evidenced by apomorphine-induced stereotypy (unpublished data).

Conversely, LID is a common condition in long-term LD treatment (Lane 2019; Gomez-Paz et al. 2025). Although the causes for LID development are not clear yet, it is believed that exogenous LD is converted to DA in serotonergic neurons and released in striatum, resulting in non-physiological pulsatile DA release, which would play an important role in LID establishment (Lane 2019; Carta et al. 2007; Carta and Tronci 2014; Cenci 2014; Stansley and Yamamoto 2015; Tronci et al. 2013; Lipari et al. 2024). When LID is studied in animal models, doses of 8–12 mg/kg of LD are usually applied to induce dyskinesia (Atanasovski et al. 2025; Liu et al. 2025). In our study, with higher doses, we did not observe the development of LID in LD-treated animals. Further studies are required to elucidate these phenomena and the possible interaction of mechanisms between LD and reserpine-induced dyskinesias.

Striatal dopamine levels are essential for proper motor control, and PD-related movement impairments emerge when a substantial loss of dopaminergic neurons in the substantia nigra leads to significant striatal dopamine depletion (Fearnley and Lees 1991; Venkatesh et al. 2025). Our findings suggest that a minimum threshold of dopamine is required to have proper motor function. Thus, effective dopaminergic treatment may not require full restoration of striatal dopamine levels, instead, it should focus on reaching functional levels. In the present study, chronic LD has only partially restored reserpine-induced DA depletion, yet this partial neurochemical effect was enough to improve motor function. This hypothesis is reinforced by the Spearman analysis showing a correlation between the levels of DA and the last catalepsy assessment. Nevertheless, the levels of DA in the striatum shown in our experiment are notably low. Possibly, the redistribution of DA within the CNS, particularly in the SNpc and other brain areas (Barriere et al. 2025), may limit the accuracy of striatal DA quantification. Other possible explanation could be the occurrence of LD conversion to DA in serotonergic neurons, which did not have a negative feedback auto-receptor to decrease DA release, promoting an uncontrolled release of DA, and a quick degradation to DOPAC and 3-MT. A possible consequence of this mechanism would be the improvement of behavior in the absence of expressive increase of dopamine in HPLC quantification (Carta et al. 2007; Carta and Tronci 2014; Stansley and Yamamoto 2015).

Previous studies of our group revealed diminished TH expression after chronic low-dose reserpine administration in STR and SNpc of rats and mice (Cunha et al. 2022; Lopes-Silva et al. 2024; Lima et al. 2021, 2025; Beserra-Filho et al. 2022; Melo et al. 2022; Lins et al. 2018; Silva-Martins et al. 2021; Dos Santos et al. 2025; Fadanni et al. 2023). Additionally, a similar reserpine protocol has been associated with decreased dopamine levels in the striatum, hippocampus, and prefrontal cortex of spontaneously hypertensive rats (Fahmy et al. 2020; Leao et al. 2021). Our findings now provide the first evidence of this dopamine depletion in mice after chronic low-dose reserpine administration. While previous studies have reported increased striatal dopamine levels at peak L-DOPA doses (Nicholas et al. 2008), our results, consistent with Obuchowicz and collaborators (Obuchowicz et al. 2003), show that this effect is not sustained 24 h post-administration.

In summary, our findings show that chronic low-dose reserpine administration induces motor impairments and striatal dopamine depletion, both of which are alleviated by LD treatment. Although the reversing effect of LD was not present in all doses and all parameters evaluated, the present results provide important support for the validity of the model, particularly when considering the acute effects of LD after the emergence of motor deficits. This closely mirrors the clinical context, in which L-DOPA treatment is typically initiated only after the onset of motor symptoms. Furthermore, under chronic administration, L-DOPA appears to further support the model by attenuating the progression of motor impairments, although it does not prevent disease progression.

## Data Availability

Data are available under request to the corresponding author.
